# Optical coherence tomography’s current clinical medical and dental applications: a review

**DOI:** 10.12688/f1000research.52031.1

**Published:** 2021-04-22

**Authors:** Saqib Ali, Saqlain Bin Syed Gilani, Juzer Shabbir, Khalid S. Almulhim, Amr Bugshan, Imran Farooq

**Affiliations:** 1 Department of Biomedical Dental Sciences, College of Dentistry, Imam Abdulrahman Bin Faisal University, Dammam, Saudi Arabia; 2 Department of Oral Biology, Islamic International Dental College, Riphah International University, Islamabad, Pakistan; 3 Department of Operative Dentistry and Endodontics, Liaquat College of Medicine and Dentistry, Karachi, Pakistan; 4 Department of Restorative Dental Sciences, College of Dentistry, Imam Abdulrahman Bin Faisal University, Dammam, Saudi Arabia; 5 Faculty of Dentistry, University of Toronto, Toronto, ON, M5G 1G6, Canada

**Keywords:** Optical coherence tomography, OCT, Imaging, Medicine, Dentistry

## Abstract

Optical coherence tomography (OCT) is a non-invasive investigative technique that is used to obtain high-resolution three-dimensional (3D) images of biological structures. This method is useful in diagnosing diseases of specific organs like the eye, where a direct biopsy cannot be conducted. Since its inception, significant advancements have been made in its technology. Apart from its initial application in ophthalmology for retinal imaging, substantial technological innovations in OCT brought by the research community have enabled its utilization beyond its original scope and allowed its application in many new clinical areas. This review presents a summary of the clinical applications of OCT in the field of medicine (ophthalmology, cardiology, otology, and dermatology) and dentistry (tissue imaging, detection of caries, analysis of dental polymer composite restorations, imaging of root canals, and diagnosis of oral cancer). In addition, potential advantages and disadvantages of OCT are also discussed.

## Introduction

Optical coherence tomography (OCT) is a non-invasive diagnostic method that can be utilized to acquire high-resolution three-dimensional (3D) images of a biological structure. ^1^ OCT has quickly gained popularity among clinicians due to several advantages that include delivering quality images with quick imaging speed. ^2^ This technique has several clinical applications that include (but are not limited to) detection of diabetic macular edema, ^3^ epithelial mapping for refractive surgery, ^4^ diagnosing and managing patients with neuro-ophthalmic conditions, ^5^ imaging of retinal microcirculation to detect hemodynamic disturbances, ^6^ detection of age associated changes in teeth, ^7^ diagnosing dental caries ^8^ and tooth wear, ^9^ and post-treatment assessments in dentistry. ^10^

The aim of this review is to summarize the clinical applications of OCT in the field of medicine and dentistry, and highlight its potential advantages and disadvantages. Google Scholar and PubMed databases were searched using keywords including “optical coherence tomography,” “OCT,” and “OCT and its clinical applications.” Our search revealed 500+ hits; all the articles published in languages other than English and conference abstracts were excluded. Only the relevant articles published in the last 10 years (2011-2021) were included to keep the information recent and contemporary. Finally, 83 articles were selected for this study and included in our review.

## Clinical applications of OCT

### Medical applications

OCT has been utilized by medical professionals extensively for diagnostic purposes and has several medical applications in ophthalmology, cardiology, otology, and dermatology. These applications will now be discussed below.

*Ophthalmology*

This technique can be used to accurately analyze choroidal thickness in the eyes under conditions such as diabetic neuropathy and macular degeneration due to aging. ^11^ In fact, it can be said with authority that this imaging tool is nowadays a routine part of ophthalmology practice to identify macular lesions. ^12^ The role of OCT is also pivotal in detecting other eye-related conditions like hyper-reflectivity due to macular telangiectasia type-2, ^13^ optic neuropathies, ^14^ and glaucomatous retinal nerve fiber layer loss. ^15^ Papilledema is a serious medical condition that leads to visual disturbances and occurs due to the optic disc's swelling because of increased intraocular pressure; ^16^^,^^17^ OCT technique can be used to measure the amount of optic disc edema in this condition. ^18^

*Cardiology*

OCT can be used for various applications in the field of cardiology. In a recent study, it has been reported that OCT-based fractional flow reserve learning can be used for enhanced treatment of intermediate coronary artery stenosis. ^19^ This technique can also be used for coronary imaging, ^20^ guiding percutaneous coronary intervention, ^21^ diagnosing myocardial infarction with non-obstructive coronary arteries, ^22^ and observing healed coronary plaques. ^23^

*Otology*

OCT can be used to accurately diagnose otology conditions such as otitis media and conductive hearing loss. ^24^ Other otology conditions where OCT has been used in the literature include diagnosis of middle ear effusions, ^25^ visualizing middle ear exudate, ^26^ observing microstructures of the middle and inner ear through an extra tympanic approach, ^27^ visualization of intra-cochlear structures for future cochlear implant surgery, ^28^ and to study endolymphatic hydrops that develops due to noise trauma. ^29^

*Dermatology*

OCT has the potential to be used to image skin layers and associated structures. In a recent study, Kato *et al.* utilized OCT to measure sweating and reported that this technique could be used successfully to measure and quantify sweating in the body. ^30^ Other OCT uses related to dermatology include characterization of micro-environment of facial pores, ^31^ aiding in the diagnosis of basal cell carcinoma, ^32^ observing the treatment response of inflammatory dermatoses, ^33^ and to detect non-melanoma skin cancers. ^34^

### Dental applications

In the past, the use of OCT was only limited to the assessment of dental hard and soft tissue morphology; however, as knowledge related to the mechanics and use of OCT increased, it led to more widespread use of OCT for different objectives. Nowadays, OCT is being used in various clinical and research applications related to dentistry. The uses of OCT in the field of dentistry are discussed in detail below.

*Tissue imaging*

Many dental tissues such as enamel and dentin can be easily visualized utilizing OCT. ^35^ The optical characteristics of enamel and dentin are different, so these structures can be distinguished from each other. Hariri *et al.* reported that OCT signals in dentin vary due to the presence of dentinal tubules and are also different from enamel. ^36^ The direction of the OCT beam to the examined surface can affect the signal intensity. The sharp slope of the enamel surface may appear bright compared to the less steep surface. ^37^ This technique has also been applied previously in various studies to detect the presence of enamel cracks. ^37^^,^^38^ Imai *et al.* visualized enamel cracks utilizing OCT and demonstrated their extension beyond dentin-enamel junction. ^38^ To detect tooth fracture in the coronal region, swept-source OCT (SS-OCT) is a useful technique. ^39^

*Detection of dental caries and demineralization of teeth*

There has been a paradigm shift in caries management recently, and this change indicates that caries should be managed conservatively. ^40^ However, it requires detection of early, un-noticeable caries activity at the earliest for it to be truly conservative. Radiography is an unreliable method of detection of carious activity. Moreover, radiography cannot distinguish properly between active and arrested lesions. ^41^ In an earlier study, it was reported that when physicians used OCT, they could detect tooth volumetric and thickness changes earlier compared with other conventional methods. ^42^ Detecting initial changes in the tooth structure could be useful in hindering the progress of dental caries. In another study, the OCT technique effectively observed the depths of carious demineralization of dentin. ^43^ Tooth demineralization can be differentiated from healthy tooth tissue by increasing light scattering in porous demineralized tooth tissue while performing OCT. ^44^ SS-OCT possesses an advantage in cases where higher resolution and penetration depth are required for cavitated caries or deeper lesion detection. ^45^ In enamel caries, the images appear brighter on grayscale OCT, and this could be the result of increased brightness due to the light reflection occurring between two homogenous structures with different refractive indices. ^46^ The demineralized mineral crystals and water in the pores cause increased reflectivity resulting in characteristic brightness in the OCT image. ^46^ SS-OCT can image dentin caries as a continuous bright area that extends from enamel into the dentin. ^44^ The signal attenuation is considered as an object parameter to differentiate between sound and demineralized enamel. ^47^ However, this attenuation can be affected by the wavelength of the incident light. The wavelength suggested to be used to detect demineralization is 1310-nm. ^48^

*Analysis of defective dental polymer composite restorations*

Conventional OCTs are considered useful in detecting the cavitated border, fracture lines, and interfacial gaps in the tooth-restoration interface. ^49^ This property appears to be due to strong reflection from the material surface in conventional OCT setups for detecting the reflective surface from the defects. ^50^ Concerning dental polymer composites, the polymer's composition could affect signal attenuation of OCT and depends on the refractive index difference between polymers resin and the filler. ^51^ OCT imaging can detect the air bubbles or void within the composite restoration ^52^ and evaluate bonding interface. ^53^ This assessment becomes easy as poorly sealed polymer composites demonstrate brightly clustered images (revealing gaps), whereas tightly fit boundaries do not exhibit too much scattering. ^54^ Ishibashi *et al.* performed a study previously and reported that SS-OCT could detect resin-based polymer composite restorations defects as it provides a much higher resolution than other conventional techniques. ^55^ The OCT technique is also a useful tool to analyze volumetric polymerization shrinkage (VPS). Sampio *et al.* performed a study to measure the VPS of different polymer composites via micro-computed tomography, and to qualitatively compare the gap formation through OCT. ^56^ It was concluded from the results of their study that while both techniques were useful, VPS assessment was largely material dependent. ^56^

*Imaging of root canals*

In an earlier *in-vitro* study, Lino *et al.* reported that SS-OCT could be used to locate the second mesiobuccal canal in maxillary molars. ^57^ Rashed *et al.* also reported similar findings in their study and reported that SS-OCT has the potential to accurately measure residual dentin thickness (RDT) and image root canals along with other internal tooth anatomic structures like pulp horns and isthmus. ^58^ The SS-OCT technique is a useful tool to accurately observe fractures of tooth roots and root canal endoscopy. ^59^ Krause *et al.* utilized OCT to monitor the pulp chamber’s roof and RDT and reported that this technique could be useful in preserving the pulp’s vitality while preparing deep cavities in dentin. ^60^

*Detection of oral cancer*

Oral cancer is one of the most fatal and prevalent cancers in the world. In India, it is the third most typical form of cancer, ^61^ and among those, 90% are oral squamous cell carcinomas. ^62^ Lee *et al.* earlier reported that OCT is a reliable technique to diagnose oral precancerous lesions with high sensitivity and specificity. ^63^ OCT has been successfully applied previously to diagnose oral malignancies in the hamster cheek pouch model. ^64^ Adegun *et al.* used OCT to diagnose epithelial dysplasia and reported that this technique could offer non-invasive method to locate the appropriate site of biopsy and thus could be useful in the early diagnosis of oral cancer. ^65^

*Other dental uses*

Peri-implantitis is the inflammation of structures around the implant, which may lead to bone resorption around the implant and eventual failure of the implant. ^66^ OCT can detect and prevent peri-implant diseases. ^67^ Kim *et al.* used OCT to measure peri-implant bone defects and reported that OCT was useful in assessing peri-implant bone levels and identifying bone defects. ^68^ Luca *et al.* used OCT to analyze bone the bone following low-level laser therapy and reported that OCT could quantitatively measure bone regeneration. ^69^ OCT is also a reliable tool to diagnose dental erosion, ^70^ assess sealing performance of resin cements, ^53^ detect invisible internal fractures of dentures non-invasively, ^71^ and monitor resin-dentin gaps. ^72^

## Advantages and disadvantages of OCT

OCT is a novel technique with diagnostic abilities used currently in different medical fields. High-resolution image acquisition of the biological tissue has enabled this modern technology to embed itself in the field of biomedical science, where researchers have been testing it for maximum possible utilization of its potential. The advantages and disadvantages of OCT have been summarized in Tables 1 and 2, respectively.

**Table 1.** Showing distinct advantages of OCT.

Advantages	References
No radiation	Shimada et al. ^50^
Non-invasiveness	Chang et al. ^73^
Repeatable	Bayer et al. ^74^
Patient-friendly	Mirzaei et al. ^75^
Gives high-resolution cross-sectional images	Hagag et al. ^76^
Rapid and safe	Chopra et al. ^77^
Portability (comes with some models)	Maloney ^78^

**Table 2.** Showing potential disadvantages of OCT.

Disadvantages	References
Low contrast images obtained for rough surfaces	Hsieh et al. ^35^
Low imaging quality due to faster imaging speed	Hsieh et al. ^35^
Increased cost	Chpora et al. ^77^
Increased skill required to operate OCT	Chopra et al. ^77^
Limited penetration depth	Israelsen et al. ^79^

## Future clinical applications

Considering the potential benefits of OCT, it can be predicted that its use in the medical field will increase further in the coming years. The combination of OCT with other techniques like scanning laser ophthalmoscopy (SLO) could prove to be useful for eye imaging when compared with the former approach alone. ^1^ The use of OCT angiography (OCTA) technique to observe human vasculature could increase in the future ^80^ as unlike SLO-based angiography methods; OCTA can provide depth information and isolation of vascular retinal ^81^ and various choroidal layers. ^82^ It will be interesting to discover the role of artificial intelligence (AI) while using OCT to diagnose skin diseases, particularly basal cell carcinoma. ^33^ Deep learning algorithms have been applied previously to the OCT technique, ^83^ but AI's role in improving OCT has not yet gained clinical maturity. It would be exciting to see the part of AI-related to OCT in the future, as it can decrease the cost and time required to train an operator. ^33^

## Conclusions

OCT is a non-invasive technique that has several clinical applications and distinct advantages. It is an explorative diagnostic method that provides clinically pertinent data, is fast, and thus it is appropriate for research and clinical practice. OCT is still an emerging technology, and considering its potential benefits, its use in medical and dental clinics should be encouraged.

## Data availability

No data is associated with this article.
