# Supplementary material for: Optical coherence tomography’s current clinical medical and dental applications: a review
Source: F1000Res. 2021 Apr 22;10:310. [Version 1] doi: 10.12688/f1000research.52031.1 (PMC8086034; doi:10.12688/f1000research.52031.1)
Supplement: Peer review history

# Peer review: Reviewer response for version 1

**DOI:** 10.5256/f1000research.55257.r83916

**Published:** 2021-04-30

**Authors:** Anand Marya

**Affiliations:** 1 Department of Orthodontics, Faculty of dentistry, University of Puthisastra, Phnom Penh, Cambodia; 2 Department of Orthodontics, Saveetha dental college, Chennai, India

First of all I would like to thank you for the editorial invitation and secondly I would like to complement the authors on their hard work and reading through it I found it to be a very well written review paper. Optical coherence tomography is slowly gaining popularity in the dental field and will surely gain more ground in future with an increased use. The authors have compiled the data associated with the uses of OCT well in this paper.

 My suggestion would be to include the types of OCT and their uses along with a brief historical perspective that would add more value to this paper. Other than that it is a very well written paper and with few additions should be indexed.

Is the review written in accessible language?

Yes

Are all factual statements correct and adequately supported by citations?

Yes

Are the conclusions drawn appropriate in the context of the current research literature?

Yes

Is the topic of the review discussed comprehensively in the context of the current literature?

Yes

Reviewer Expertise:

Orthodontics, dental diagnosis, dentistry

I confirm that I have read this submission and believe that I have an appropriate level of expertise to confirm that it is of an acceptable scientific standard.

# Peer review: Reviewer response for version 1

**DOI:** 10.5256/f1000research.55257.r83915

**Published:** 2021-04-29

**Authors:** A. Thirumal Raj

**Affiliations:** 1 Department of Oral Pathology and Microbiology, Sri Venkateswara Dental College and Hospital, Chennai, Tamil Nadu, India

The article is interesting and summarizes various medical and dental applications of the optical coherence tomography (OCT) technique. I have reviewed it and would like to have it indexed. Few suggested improvements from my side are as follows:

1. The introduction section can be expanded further.
2. More medical applications of the OCT technique can be included.
3. Future work section can be more detailed.
4. More references can be added, which would help to expand different sections.

Is the review written in accessible language?

Yes

Are all factual statements correct and adequately supported by citations?

Yes

Are the conclusions drawn appropriate in the context of the current research literature?

Yes

Is the topic of the review discussed comprehensively in the context of the current literature?

Yes

Reviewer Expertise:

Oral pathology

I confirm that I have read this submission and believe that I have an appropriate level of expertise to confirm that it is of an acceptable scientific standard.

# Peer review: Reviewer response for version 1

**DOI:** 10.5256/f1000research.55257.r83913

**Published:** 2021-04-27

**Authors:** Farooq Ahmad Chaudhary

**Affiliations:** 1 School of Dentistry, Shaheed Zulfiqar Ali Bhutto Medical University, Islamabad, Pakistan

The article deals with the clinical applications of optical coherence tomography. It's well-written and I just have few minor comments:

1. Introduction could be expanded to include basics of the OCT technique.
2. An image showing the working principle of the OCT technique could be useful in the introduction section.
3. It would be easier for the readers if the article contained a flowchart of the search strategy. 
4. Dental applications are well-written, but a little more explanation of medical applications could attract more readers.

Is the review written in accessible language?

Yes

Are all factual statements correct and adequately supported by citations?

Yes

Are the conclusions drawn appropriate in the context of the current research literature?

Yes

Is the topic of the review discussed comprehensively in the context of the current literature?

Yes

Reviewer Expertise:

Dentistry, Oral Health, and Preventive Dentistry

I confirm that I have read this submission and believe that I have an appropriate level of expertise to confirm that it is of an acceptable scientific standard.
